# Complement C4-deficient mice have a high mortality rate during PTZ-induced epileptic seizures, which correlates with cognitive problems and the deficiency in the expression of Egr1 and other immediate early genes

**DOI:** 10.3389/fncel.2023.1170031

**Published:** 2023-05-10

**Authors:** Tatyana Veremeyko, Rongcai Jiang, Mingliang He, Eugene D. Ponomarev

**Affiliations:** ^1^Department of Biomedical Sciences, Jockey Club College of Veterinary Medicine and Life Sciences, City University of Hong Kong, Hong Kong, Hong Kong SAR, China; ^2^Department of Biology, School of Sciences and Humanities, Nazarbayev University, Astana, Kazakhstan; ^3^Chinese University of Hong Kong Joint Laboratory of Bioresources and Molecular Research of Common Diseases, Kunming Institute of Zoology, Chinese Academy of Sciences, Kunming, China; ^4^Department of Neurosurgery, Tianjin Medical University General Hospital, Tianjin, China; ^5^Tianjin Neurological Institute, Key Laboratory of Post Neuro-Injury Neuro-Repair and Regeneration in Central Nervous System, Ministry of Education and Tianjin City, Tianjin, China

**Keywords:** complement, C4, epilepsy, Egr1, cognitive functions, BDNF, neuroinflammation, immediate early gene (IEG)

## Abstract

Complement system plays an important role in the immune defense against pathogens; however, recent studies demonstrated an important role of complement subunits C1q, C4, and C3 in normal functions of the central nervous system (CNS) such as non-functional synapse elimination (synapse pruning), and during various neurologic pathologies. Humans have two forms of C4 protein encoded by C4A and C4B genes that share 99.5% homology, while mice have only one C4B gene that is functionally active in the complement cascade. Overexpression of the human C4A gene was shown to contribute to the development of schizophrenia by mediating extensive synapse pruning through the activation C1q-C4-C3 pathway, while C4B deficiency or low levels of C4B expression were shown to relate to the development of schizophrenia and autism spectrum disorders possibly via other mechanisms not related to synapse elimination. To investigate the potential role of C4B in neuronal functions not related to synapse pruning, we compared wildtype (WT) mice with C3- and C4B- deficient animals for their susceptibility to pentylenetetrazole (PTZ)- induced epileptic seizures. We found that C4B (but not C3)–deficient mice were highly susceptible to convulsant and subconvulsant doses of PTZ when compared to WT controls. Further gene expression analysis revealed that in contrast to WT or C3-deficient animals, C4B-deficient mice failed to upregulate expressions of multiple immediate early genes (IEGs) Egrs1-4, c-Fos, c-Jus, FosB, Npas4, and Nur77 during epileptic seizures. Moreover, C4B-deficient mice had low levels of baseline expression of Egr1 on mRNA and protein levels, which was correlated with the cognitive problems of these animals. C4-deficient animals also failed to upregulate several genes downstream of IEGs such as BDNF and pro-inflammatory cytokines IL-1β, IL-6, and TNF. Taken together, our study demonstrates a new role of C4B in the regulation of expression of IEGs and their downstream targets during CNS insults such as epileptic seizures.

## 1. Introduction

The complement system consists of more than 30 proteins forming an enzymatic activating cascade that is initiated by various “danger signals” such as pathogens, cellular debris, apoptotic bodies, and blood coagulation processes (Foley and Conway, 2016; Reis et al., 2019). Specifically, complement may be activated via the classical, lectin, and alternative pathways, or an extrinsic blood coagulation cascade (Foley and Conway, 2016; Chen et al., 2022). The classical pathway is initiated by antigen–antibody complexes that bind C1q, leading to the proteolytic cleavage of C2 into C2a and C2b fragments, and then cleavage of C4 into C4a and C4b fragments. Fragments C4b and C2a bind to each other to form the C4bC2a dimer complex (also referred to as C3-convertase), which then cleaves C3 into C3a and C3b fragments. Subsequently, fragment C3b and C4bC2a dimer form C5-convertase (C3bC4bC2a trimer) that further cleaves C5 leading to the formation of membrane attack complex. In the lectin pathway, C4 is cleaved by the MASP-2 enzyme into C4a and C4b (Wallis et al., 2007). Thus, both classical and lectin pathways lead to C4 activation, and C4b could further activate downstream C3, or C4b could just act as an opsonin. The liver is the dominant source of most complement proteins in the plasma or serum, except for C1q, which is mainly synthesized by immune cells. In the central nervous system (CNS), several complement components, including C1q, C4, and C3 are synthesized by astrocytes, oligodendrocytes, neurons, and microglia (Cho, 2019; Schartz and Tenner, 2020; Peoples and Strang, 2021; Chen et al., 2022). In the normal CNS, microglia produce C1q, which then recruits C3 to axons via activation of C4 in the classical pathway and thus marks unfunctional synapses for elimination by microglia via microglial complement receptor 3 (CR3) that specifically binds to C3b (Stevens et al., 2007; Paolicelli et al., 2011; Cho, 2019; Gomez-Arboledas et al., 2021). It was recently demonstrated that the complement system is involved in the pathogenesis of many types of neurologic diseases including traumatic brain injury, Alzheimer’s disease, amyotrophic lateral sclerosis, Parkinson’s disease, and multiple sclerosis (Schartz and Tenner, 2020; Gomez-Arboledas et al., 2021; Chen et al., 2022). Genome-wide association studies demonstrated a strong association between human *C4* gene polymorphisms and schizophrenia (SZ) (Sekar et al., 2016).

Complement C4 protein is a non-enzymatic, but essential component of C3 and C5 convertases that plays a key role in the propagation of classical and lectin complement activation pathways (Blanchong et al., 2001; Peoples and Strang, 2021). Human *C4* genes are located in MHC (HLA) class III locus on chromosome 6 and, similar to other MHC genes, *C4* genes have a high level of polymorphism. Human C4 protein has two isoforms encoded by *C4A* and *C4B* genes, which share 99.5% homology, but have distinct affinities to their carbohydrate targets on cell/pathogen surface and their patterns of expression (Blanchong et al., 2001). More specifically, *C4A* is induced or enhanced by IFNγ during inflammation, while *C4B* is constitutively present in high concentrations in plasma or serum (Kulics et al., 1990; Blanchong et al., 2001). Mice have *Slp* and *C4B* homologs for human *C4A* and *C4B* genes, respectively. Unlike human C4 genes, *Slp* and *C4B* share only 95% homology and the *Slp* gene is not functionally active in complement enzymatic cascade having *C4B* as the single functional gene for complement C4 component in mice (Blanchong et al., 2001).

The elevated level of *C4A* and, to lesser extent, *C4B* were associated with SZ (Sekar et al., 2016), which was also investigated in mouse models with overexpression of human *C4A* or mouse *C4B* genes that mediated excessive synapse elimination and behavior changes (Comer et al., 2020; Druart et al., 2021; Yilmaz et al., 2021). On the other hand, in humans, low levels, or deficiency in *C4B* expression were associated with a high risk of SZ, autism spectrum disorders (ASD), and certain autoimmune diseases (Mayilyan et al., 2008; Mostafa and Shehab, 2010; Zhou et al., 2021). It was demonstrated that *C1q*-, *C4B*-, and *C3*- deficient mice have a higher density of synapses and these elevated densities of synapses were comparable for these three groups of animals (Stevens et al., 2007; Sekar et al., 2016; Cho, 2019; Yilmaz et al., 2021). Given the fact that C1q and C4 are upstream of C3 activation in the classical complement activation pathway (Peoples and Strang, 2021; Chen et al., 2022), these results are not surprising in terms of insufficient synapse pruning in these animals when compared to wild-type (WT) controls. However, it is not clear why C4-, but not C3-, deficiency and/or polymorphism is associated with neurological disorders such as SZ and ASD in humans.

The role of complement in the development of epilepsy appeared to be even more complex and controversial. It was demonstrated that *C1q*-deficient mice are prone to behavior seizures and have a higher baseline level of neuronal electric activity, which was due to the elevated density of neuronal processes as a consequence of insufficient synaptic pruning via C1q-C3-CR3 mechanism (Chu et al., 2010). Above mentioned study showed the anti-epileptic role of C1q. On the other hand, it was demonstrated that C1q and C3 contributed to epilepsy in several mouse models (Holden et al., 2021; Jiang et al., 2021), while elevated plasma level of C4 was found to be a marker of uncontrolled seizures in humans (Kopczynska et al., 2018). Finally, the classical C1q-C4-C3 pathway of complement activation was demonstrated to occur during epileptic seizures (Chu et al., 2010). Thus, the differential role of C4 vs. C3 in the development of seizures or other pathologies such as SZ and ASD were not extensively studied so far in terms of the basic functions of C4 in the CNS.

In this study, we hypothesize that C4 plays an additional role in neuronal cell functions besides its established role as an upstream activator of C3 in the classical pathway that is required for synapse pruning. To test our hypothesis, we induced epileptic seizures in WT, C4B-, and C3- deficient mice using pentylenetetrazole (PTZ), a GABA_*A*_ receptor antagonist (MacDonald and Barker, 1977), a well-known convulsant that induces seizures in various species from humans to mice. We found that C4B-, but not C3-, deficient mice were highly sensitive to the standard convulsant dose of PTZ (80 mg/kg) with very severe seizures leading to a 100% mortality rate when compared to WT controls. Moreover, a subconvulsant dose of PTZ (50 mg/kg) induced severe seizures with a 60% mortality rate in C4B-deficient mice but failed to do so in C3-deficient and WT mice. Further gene expression analysis revealed that in contrast to WT or C3-deficient groups, C4B-deficient mice were unable to upregulate mRNA for several immediate early genes (IEGs): *Egr1*, *Egr2*, *Egr3*, *Egr4*, *c-Fos*, *c-Jun*, *FosB*, *Npas4*, *Nur77*, as well as neurotrophic factor *BDNF* and major pro-inflammatory cytokines *IL-1*β, *IL-6*, and *TNF*, which are downstream of IEGs. Our data indicate that in contrast to WT or C3-deficient mice, C4B-deficient animals could not adequately respond to CNS insults or stress conditions such as epileptic seizures, which likely lead to extensive dysregulation of neuronal functions and a high mortality rate.

## 2. Materials and methods

### 2.1. Mice

C57BL/6 (B6; WT), *C3-*deficient *B6.129S4-C3tm1Crr/J* (C3^—/—^), and *C4B*-deficient *B6.129S4-C4btm1Crr/J* (C4B^––/––^) mice were purchased from Jackson Laboratories (Bar Harbour, ME, USA), and then maintained and experimentally manipulated at the Laboratory Animal Service Centre at the Chinese University of Hong Kong (Shatin, Hong Kong). The study was performed following the recommendations of the ARRIVE guidelines^1^ and all experimental procedures were approved by a health and food department of the government of Hong Kong, the Chinese University of Hong Kong, and the City University of Hong Kong Animal Experimentation Ethics Committees. All mice were housed in a temperature-controlled environment with an automatic 12 h light/dark cycle and were fed *ad libitum*. Genotyping of C3^–/–^ and C4B^–/–^ animals was performed in comparison with WT controls by standard PCR using primer sets and the protocols provided by Jackson Laboratory. In addition to genotyping, the phenotype of C3- and C4B- deficient mice was further confirmed by examining the levels of C3 and C4B in mouse serum using western blotting as shown in Supplementary Figures 1, 2, respectively. The blood samples were collected from retro-orbital sinuses of anesthetized animals using glass capillaries as we did in our earlier studies (Starossom et al., 2014) and allowed to be coagulated for 1 h at 37°C. The serum samples were obtained by centrifugation of coagulated blood samples according to standard protocol (Starossom et al., 2015).

### 2.2. PTZ-induced seizures

Pentylenetetrazole (PTZ; also known as pentylenetetrazole, leptazol, metrazol, pentetrazol, pentamethylenetetrazol, corazol, cardiazol, and deumacard) was purchased from Sigma (Cat. No. P6500, Sigma, St. Louis, MO, USA), dissolved in PBS, and was injected intraperitoneally (i.p.) in 8–12-week-old WT, C3^–/–^ or C4B^–/–^ female mice at a dose of 80 mg/kg (convulsant dose) or 50 mg/kg (subconvulsant dose) as we did earlier in our previous study (Kopeikina et al., 2020). We used only female animals in our studies since it was shown that WT females are more susceptible to seizures than males in case of subconvulsant (50–60 mg/kg) dosage of PTZ (Medina et al., 2001). At the same time, there was no sex-specific difference for seizure susceptibility for convulsant dosage of PTZ [70–80 mg/kg; (Medina et al., 2001) and unpublished observation]. The mice were video-recorded for 10 min post-PTZ administration to analyze the latency of severe seizure development, and to determine the maximal clinical epileptic scores developed over the 10-min period. Clinical epileptic scores were assigned as we previously described in our study: (0) normal behavior, no abnormality; (1) facial twitching, restless behavior; (2) myoclonic body jerks; (3) fore limb clonus; (4) rearing, tonic seizure, falling on their side; (5) tonic-clonic seizure, falling on their backs, wild rushing, jumping; and (6) death. The seizure latency was measured as the delay time until the start of tonic-clonic seizures of epileptic score 3 or above during the 10-min period post-PTZ administration. If tonic-clonic seizure did not appear within the 10-min period, we followed the mice for 4h to monitor recovery and recorded the latency time as 10 min.

### 2.3. Gene expression analysis by real-time RT PCR

For RNA isolation from brain tissues, unmanipulated WT, C3^–/–^ or C4B^–/–^ mice, or alive WT, C3^–/–^ or C4B^–/–^ animals 9 ± 1 min post-PTZ injection were euthanized by swift decapitation. Brain frontal cortexes were dissected as we did earlier in our studies (Kopeikina et al., 2020; Strekalova et al., 2021a,b), washed with PBS, frozen in dry ice, stored for 1–2 weeks at −80°C till further processing, and then transferred to the laboratory in dry ice. Subsequently the samples were unfrozen, homogenized using a 10 ml glass/Teflon Wheaton tissue grinder (Cat. No. 358039, DWK Life Sciences LLC, Millville, NJ, USA), and lysed using QIAzol Lysis Reagent (Cat. No. 79306, Qiagen, Hilden, Germany) as described in our previous studies (Dukhinova et al., 2019; Veremeyko et al., 2019b; Kopeikina et al., 2020). Further RNA purification was performed using DNase digestion and miRNeasy Mini Kit from Qiagen (Cat. No. 217004). Real-time RT PCR was performed using ABI ViiA 7 and ABI QuantStudio 7 (QS7) Flex Systems. The primer sets are summarized in Table 1. Relative expression levels were calculated using the ΔΔC_*T*_ method and normalized to the expression of the GADPH housekeeping gene and then to the expression of a control sample, which was defined as 1 as we did in our previous studies (Gorlova et al., 2019; Pavlov et al., 2019; Veremeyko et al., 2019a).

**TABLE 1:** Primer sequence for gene expression analysis.

Gene	Primer	Sequence
GADPH	Forward	5′-ATGACCACAGTCCATGCCATC -3′
	Reverse	5′-GAGCTTCCCGTTCAGCTCTG -3′
*Egr1*	Forward	5′-GCAGCGCCTTCAATCCTCAAG-3′
	Reverse	5′-GCTCACGAGGCCACTGACTAG-3′
*Egr2*	Forward	5′- CCCTTTGACCAGATGAACGGAG-3′
	Reverse	5′-AAGCTACTCGGATACGGGAGATC-3′
*Egr3*	Forward	5′- CGACTCGGTAGCCCATTACAATC -3′
	Reverse	5′-GGGCTTCTCGTTGGTCAGAC-3′
*Egr4*	Forward	5′-GCTTCTTCATCCAGGCGGTTC-3′
	Reverse	5′-GCCCAAGATGCCAGACATGAG-3′
*c-Fos*	Forward	5′-ATCGGCAGAAGGGGCAAAGTAG-3′
	Reverse	5′-GCAAGGGTAACAGCGGTGAA-3′
*c-Jun*	Forward	5′-GTTGCG GCCGCGAAACTT-3′
	Reverse	5′-CATTGCCCTCGAGCCCTG-3′
*FosB*	Forward	5′-GGGAGCTGACAGATCGACTTC-3′
	Reverse	5′-CAAACTCCAGGCGTTCCTTCTC-3′
*Nur77*	Forward	5′-GCTAGAGTCTGCCTTCCTGGAAC-3′
	Reverse	5′-GCACACTGCAGCTGGTGTAG-3′
*Npas4*	Forward	5′-GGAGGCTGGACATGGATTTACTG-3′
	Reverse	5′-CTGCCTGGGTGTCTTCAGAG-3′
*Arc*	Forward	5′- GTGATCCTGCAGATTGGTAAGTGC -3′
	Reverse	5′- ACGTGCATCTCACGCTTGAC -3′
*BDNF*	Forward	5′-GTCATCGAAGAGCTGCTGGATG -3′
	Reverse	5′-CTCCAAAGGCACTTGACTGCTG -3′
*GABRQ*	Forward	5′-CACGGTCCTCACCACCATTC -3′
	Reverse	5′-CCGCATATGTGAGTCGATGGTAG -3′
*GABRA2*	Forward	5′-CTTCTGACTCCGTTCAGGTTGC -3′
	Reverse	5′-GCAAGGCAGATAGGTCTGAATCAC -3′
*IL-1B*	Forward	5′- CTTCCAGGATGAGGACATGAGCAC -3′
	Reverse	5′-TCATCATCCCATGAGTCACAGAGG -3′
*IL-6*	Forward	5′-CCTTCTTGGGACTGATGCTGGTG-3′
	Reverse	5′- AGGTCTGTTGGGAGTGGTATCCTC-3′
*TNF*	Forward	5′-AGCCGATGGGTTGTACCTTG- 3′
	Reverse	5′- GTGGGTGAGGAGCACGTAGTC -3′
*PSD95*	Forward	5′-TCTGTGCGAGAGGTAGCAGA-3′
	Reverse	5′-AAGCACTCCGTGAACTCCTG-3′
*Syn1*	Forward	5′-CCGCCAGCTGCCTTC-3′
	Reverse	5′-TGCAGCCCAATGACCAAA-3′
*Mecp2*	Forward	5′-GCCGATCTGCTGGAAAGTATGATG-3′
	Reverse	5′-CCTCTCCCAGTTACCGTGAAGTC-3′

### 2.4. Western blotting

Western blot analysis was performed according to a standard protocol as previously reported in our earlier studies (Veremeyko et al., 2018, 2019a; Dukhinova et al., 2019). The following antibodies were used for β-Actin (Cell Signalling, Gene Company Limited, Chai Wan, Hong Kong, Cat. No. 4967, dilution 1:10000), Egr1 (Cell Signalling, Cat. No. 4154, dilution 1:1000), C3 (Invitrogen, Waltham, MA, USA, Cat. No. PA1-29715, dilution 1:1000), C4 (Abbexa, Cambridge, U.K., Cat. No. abx129540, dilution 1:1000), and PSD95 (Cell Signalling, Cat. No. 3450, dilution 1:1000). As a secondary antibody, we used various anti-rabbit or anti-goat antibodies conjugated with alkaline phosphatase (purchased from Abcam, Cambridge, U.K.). For quantitative EGR1 and PSD95 protein level analysis, β-Actin was used as a loading control. For analysis of plasma levels of α- and β-chains of C3 (Supplementary Figure 1) and γ-chain of C4 (Supplementary Figure 2), the total protein concentration in serum samples was measured by Bradford assay (Sigma, Cat. No. B6916), equal quantities of serum proteins were loaded on polyacrylamide gels (ThermoFisher Inc., Life Technologies, Carlsbad, CA, USA), and Ponceau S (Abcam, Cat. No. ab270042) staining was used as a loading control (Supplementary Figures 1, 2).

### 2.5. Behavior tests

To test motor functions, we utilized a standard five-lane mouse Rotarod set-up (Med Associates Inc., Fairfax, VT, USA) with programmed accelerated rotation (4–40 rpm). For testing, the animals were first trained to stay on the rotating rod at a low speed of 4 rpm on day 1 for 10–15 min, and then the total time that an animal can stay on the accelerated rod before falling was used to measure the motor functions (coordination and balance) on days 1–5 as similar as it was described earlier in our previous study (Kopeikina et al., 2020).

To test cognitive functions in mice, we used a custom-made Barns maze set-up. Particularly, we used a white-color circular platform (92 cm in diameter) with 20 equally spaced holes (5 cm in diameter; the distance is 7.5 cm between holes) as described in our earlier studies (Dukhinova et al., 2018, 2019). The bright lighting and fan were turned on during testing to stimulate mice to locate one hole with the escape box attached to it. Mice were trained to locate a hole where they can escape from the platform into the escape box during three daily training sessions. The platform was randomly rotated and washed with 70% ethanol before each training session for each mouse. Latency to escape was scored manually on days 1–4 during the training sessions (three training sessions per day for each mouse). On day 5, the final trial was performed, where animals could not escape the platform with the escape hole closed and their time to reach the area of the closed hole was evaluated using Noldus EthoVision XT (v. 11) software (Wageningen, the Netherlands).

### 2.6. Statistics

The results are presented as box and whisker plots or mean ± standard error (SE) with overlayed dot plots. One-way or two-way ANOVA with or without the Tukey *post-hoc* tests were used to determine statistical significance for experiments with three or more experimental groups. *P*-values of less than 0.05 were considered significant. GraphPad Prism software (Boston MA, USA) was used for the creation of the graphs and further statistical analysis.

## 3. Results

### 3.1. CB4-, but not C3-, deficient mice develop very severe seizures with a 100% mortality rate

**FIGURE 1:**
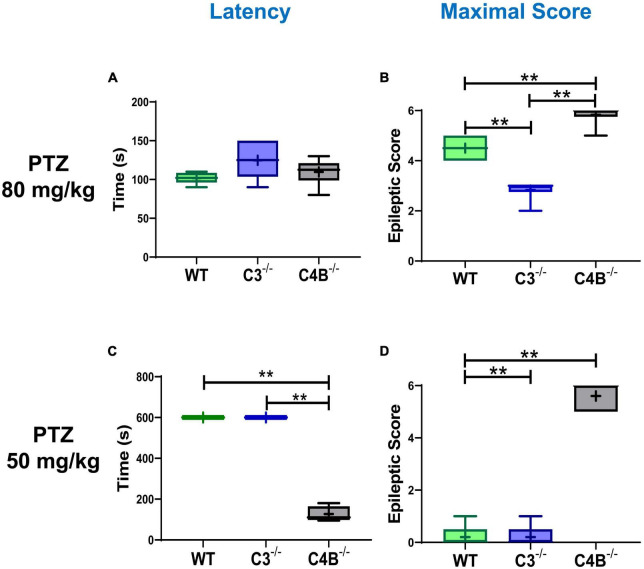
Comparison of seizure latency and severity in WT, C3- and C4B-deficient mice. Groups of WT, or C3^–/–^, or C4B^–/–^ mice were injected i.p. with convulsant (80 mg/kg) **(A,B)** or subconvulsant (50 mg/kg) **(C,D)** doses of PTZ, and the seizure latency **(A,C)**, and maximal epileptic scores **(B,D)** were measured as described in Section “2. Materials and methods”. The median with 10%/90% percentiles of individual mice is shown in a box and whisker plot with the mean value indicated by “+”. The indicated differences were statistically significant, as determined by one-way ANOVA followed by Tukey *post-hoc* tests [**p < 0.01 for comparisons between two groups; **(A,B)** *n* = 6 mice; **(B)** *p* < 0.0001, *F*(2,15) = 64.21; **(C,D)** *n* = 5; **(C)** *p* < 0.0001, *F*(2,12) = 831.7; **(D)** *p* < 0.0001, *F*(2,12) = 208.3].

To determine the differential role of C4 vs. C3 proteins during epileptic insult, we induced seizures in WT, C3^–/–^, and C4B^–/–^ mice using the standard convulsant dose of PTZ of 80 mg/kg (Kopeikina et al., 2020). All three experimental groups developed seizures, with a comparable mean latency time of 102 s for WT and 110 s for C4B^–/–^ mice (Figure 1A). There was a trend of a slight increase in mean latency for C3-deficient mice (125 s), but it was not statistically significant (Figure 1A). However, when we analyzed the severity of seizures using epileptic scores, we found that C4B-deficient mice had very severe seizures with maximal epileptic scores of 5–6 developed within 10 min period post-PTZ administration (Figure 1B), and 100% mortality rate within 6–12 min period post-PTZ-administration (Table 2). At the same time, WT mice had maximal epileptic scores of 4–5 (Figure 1B) with a mortality rate of 0% (Table 2). Quite surprisingly in contrast to C4B-deficient mice, C3**^–/–^** mice had lower maximal epileptic scores of 2–3 when compared to WT mice; however, one C3-deficient mouse out of six died 28 min post-PTZ injection comprising a 16% mortality rate (Table 2). When we injected a subconvulsant dose of PTZ [50 mg/kg (Kopeikina et al., 2020)], we found, as it was expected, that WT mice did not develop tonic-clonic seizures with scores 3 or above over 10 min period with maximal epileptic scores 0–1 (no symptoms or facial twitching) and 0% mortality (Figures 1C, D; Table 2). Interestingly C3-deficient mice showed the same results as the WT group with a maximal score of 0–1 and 0% mortality rate (Figures 1C, D; Table 2). However, C4B-deficient mice developed severe seizures with ∼100 s latency to score 3 or above (Figure 1C), maximal epileptic scores 5–6 (Figure 1D), and a 60% mortality rate (Table 2). Thus, these data indicate that C4B-, but not C3-, deficient animals were very susceptible to seizure development with convulsant and subconvulsant doses of PTZ.

**TABLE 2:** The mortality rate in wild-type (WT), C3-, and C4-deficient mice with epileptic seizures induced by convulsant (80 mg/kg) and sub-convulsant (50 mg/kg) dosages of PTZ^1^.

PTZ dosage	80 mg/kg			50 mg/kg		
Mouse genotype	WT	C3^–/–^	C4^–/–^	WT	C3^–/–^	C4^–/–^
Mortality rate, % (m/n)	0% (0/6)	16%^2^ (1/6)	100%^3^ (6/6)	0% (0/5)	0% (0/5)	60%^3^ (3/5)

^1^Epileptic seizures were induced by i.p. injection of PTZ in WT, C3^–/–^, and C4^–/–^ mice at the dosage of 80 mg/kg or 50 mg/kg, and mice were monitored for 4h for their survival as described in Section “2. Materials and methods”.

^2^Died 28 min post-PTZ injection.

^3^Died 6–12 min post-PTZ injection.

### 3.2. C4-deficient mice fail to upregulate immediate early genes during epileptic seizures

**FIGURE 2:**
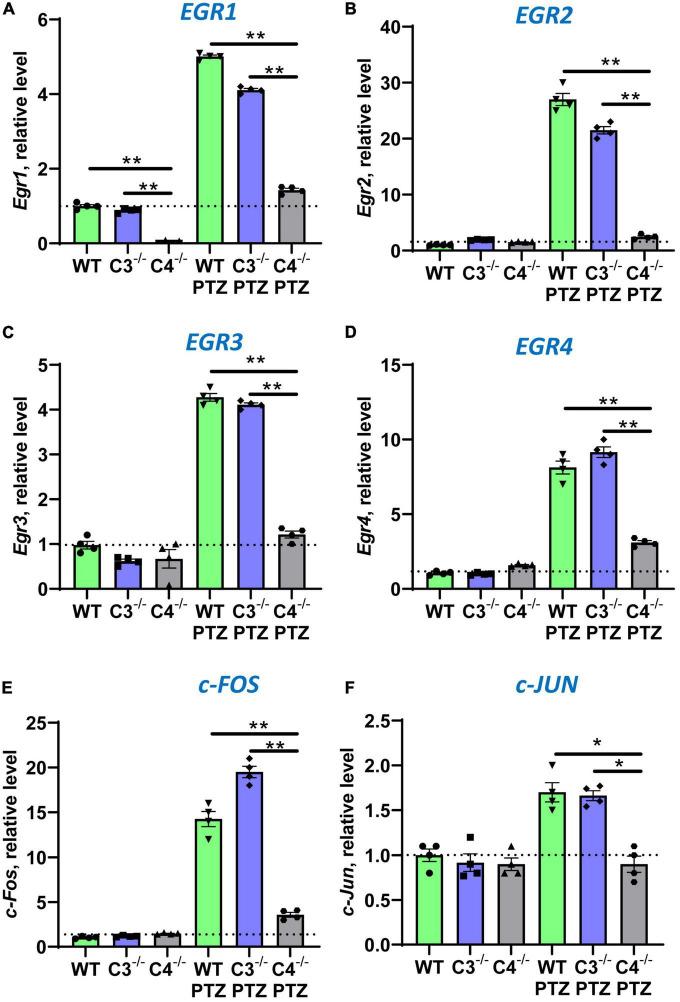
Analysis of the expression of *Egr1*, *Egr2*, *Egr3*, *Egr4*, *c-Fos*, and *c-Jun* immediate early genes in the brain cortex of unmanipulated mice or mice with PTZ-induced seizures in groups of WT, or C3^–/–^, or C4B^–/–^ animals. RNA was isolated from brain cortexes of unmanipulated WT, or C3^–/–^, or C4B^–/–^ mice (left three bars in each panel) vs. WT, or C3^–/–^, or C4B^–/–^ mice 9 ± 1 min post-PTZ administration (right three bars in each panel), and the expressions of *Egr1* **(A)**, *Egr2* **(B)**, *Egr3* **(C)**, *Egr4* **(D)**, *c-Fos* **(E)**, and *c-Jun* **(F)** were analyzed by real-time RT PCR as described in the Section “2. Materials and methods”. Mean ± standard error (SE) with overlayed dot plots is shown. The indicated differences were statistically significant, as determined by one-way ANOVA followed by Tukey *post-hoc* test (*p < 0.05, **p < 0.01 for comparisons between two indicated groups; *n* = 4 mice).

**FIGURE 3:**
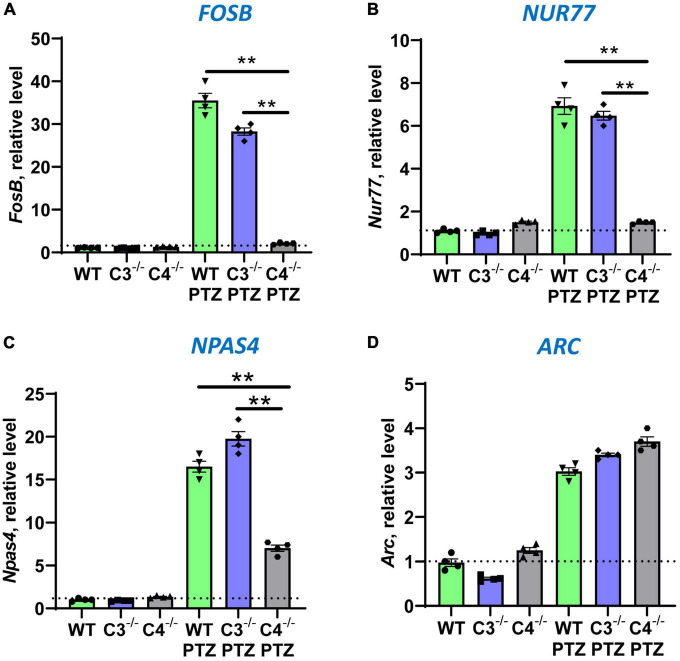
Analysis of the expression of *FosB*, *Nur77*, *Npas4*, and *Arc* immediate early genes in brain cortexes of unmanipulated mice or mice with PTZ-induced seizures in groups of WT, or C3^–/–^, or C4B^–/–^ animals. RNA was isolated from brain cortexes of unmanipulated WT, or C3^–/–^, or C4B^–/–^ mice (left three bars in each panel) vs. WT, or C3^–/–^, or C4B^–/–^ mice 9 ± 1 min post-PTZ administration (right three bars in each panel), and the expressions of *FosB* **(A)**, *Nur77* **(B)**, *Npas4* **(C)**, and *Arc* **(D)** were analyzed by real-time RT PCR as described in Section “2. Materials and methods”. Mean ± standard error (SE) with overlayed dot plots is shown. The indicated differences were statistically significant, as determined by one-way ANOVA followed by Tukey *post-hoc* test (**p < 0.01 for comparisons between two indicated groups; *n* = 4 mice).

It is well known that during epileptic seizures neuronal cells upregulate IEGs within minutes (Kalinina et al., 2022). IEGs are responsible for many neuronal functions including their activation, long-term potentiation (LTP), memory, and CNS repair (Kiessling and Gass, 1993; Dragunow, 1996; Gallo et al., 2018). We hypothesized that C4-deficient mice abnormally respond to stress conditions such as seizures, which is reflected in the abnormal pattern of IEG expression in the CNS. To test our hypothesis, we performed selective profiling of ten IEGs at a baseline level in unmanipulated mice and 9 ± 1 min post-PTZ injection in the brain of WT, C3- and C4B-deficient mice. Time-point of 9 ± 1 min was selected to ensure that all tested animals including C4B^–/–^ are alive. We found that out of 10 tested IEGs, nine were expressed in the brain of WT, C3-, and C4B-deficient mice at comparable baseline levels (Figures 2, 3; WT, C3^–/–^, C4^–/–^), except for Egr1 that was significantly reduced in the brain of C4B-deficient mice when compared to C3^–/–^ and WT groups (Figure 2A; WT, C3^–/–^, C4^–/–^). Nine minutes post-PTZ injection, WT and C3^–/–^ mice substantially upregulated (from ∼2 to 40-fold changes) all ten tested IEGs: *Egrs1-4*, *c-Fos*, *c-Jun* (Figure 2), *FosB*, *Nur77, Npas 4* and *Arc* (Figures 3A-D); however, C4B^–/–^ mice could not upregulate expression of nine of ten tested IEGs to the level of WT or C3^–/–^ animals except for *Arc*, which was upregulated in C4B^–/–^ mice to the level comparable to WT and C3^–/–^ mice (Figure 3D). Thus, these data indicate that after PTZ administration C4B^–/–^, but not WT or C3^–/–^, mice could not substantially upregulate nine out of ten tested IEGs: *Egrs1-4*, *c-Fos*, *c-Jun*, *ForB*, *Naps4*, and *Nur77*.

### 3.3. C4-deficient mice fail to upregulate CNS repair and pro-inflammatory genes

**FIGURE 4:**
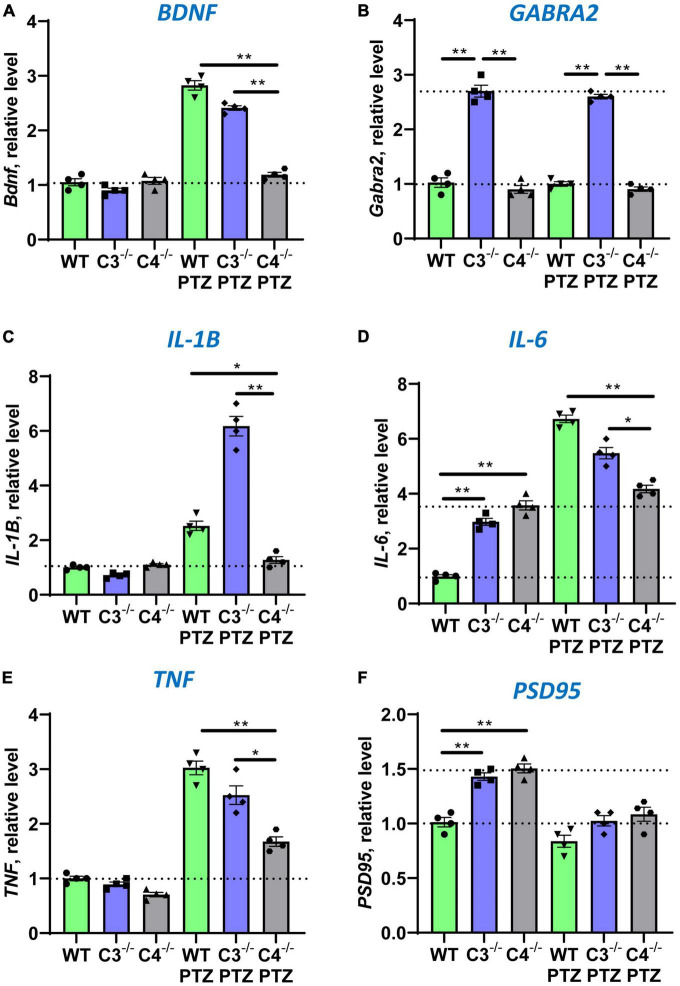
Analysis of the expression of *BDNF*, *GABRA2*, *IL-1*β, *IL-6*, *TNF*, and *PSD95* in the brain cortex of unmanipulated mice or mice with PTZ-induced seizures in groups of WT, or C3^–/–^, or C4B^–/–^ animals. RNA was isolated from brain cortexes of unmanipulated WT, or C3^–/–^, or C4B^–/–^ mice (left three bars in each panel) vs. WT, or C3^–/–^, or C4B^–/–^ mice 9 ± 1 min post-PTZ administration (right three bars in each panel), and the expressions of *BDNF* **(A)**, *GABRA2* **(B)**, *IL-1*β **(C)**, *IL-6* **(D)**, *TNF* **(E)**, and PSD95 **(F)** were analyzed by real-time RT PCR as described in Section “2. Materials and methods”. Mean ± standard error (SE) with overlayed dot plots is shown. The indicated differences were statistically significant, as determined by one-way ANOVA followed by Tukey *post-hoc* test (*p < 0.05, **p < 0.01 for comparisons between two groups; *n* = 4 mice).

In the next series of experiments, we investigated the expression of other genes in the CNS, which are downstream of IEGs. It was demonstrated that *Npas4* directly regulates *BDNF*, an important neurotrophic factor involved in neuronal protection and CNS repair (Fu et al., 2020). Indeed, we found that C4B-deficient mice could not upregulate *BDNF* in the brain after PTZ injection when compared to WT and C3-deficient mice (Figure 4A). It was also demonstrated that *Egr1* directly regulate expression of *GABRA2* and *GABRQ* genes that are involved in inhibitory synapse activity by forming subunits for GABA_*A*_ receptors in post-synaptic membranes (Mo et al., 2015). Since C4B-deficient mice had a lower level of *Egr1* at baseline level in the CNS and it was not substantially upregulated in the brain after PTZ injection when compared to WT and C3^–/–^ controls (Figure 2A), we investigated the level of *GABRA2* and *GABRQ* genes in WT vs. C3^–/–^ vs. C4B^–/–^ mice. We did not find differences in *GABRQ* expression levels (not shown), but C3^–/–^ mice had a 2.8-fold higher level of *GABRA2* at the baseline level in the brain, which was not altered after PTZ injection (Figure 4B), when compared to WT and C4B^–/–^ mice. An elevated level of *GABRA2* in the brain of C3-deficient mice might explain lower maximal epileptic scores in these mice after injection of PTZ (Figure 1B), which is a GABA_*A*_ receptor antagonist. Finally, we investigated the expression of three major pro-inflammatory cytokines IL-1β, IL-6, and TNF in the CNS at the baseline level and post-PTZ injection in three experimental groups. Two IEGs c-Fos and c-Jun form dimers comprising transcription factor AP-1, which is known to induce the expression of pro-inflammatory cytokines in innate immune cells (Guha and Mackman, 2001; Veremeyko et al., 2019b). Since C4B-deficient mice failed to upregulate both c-Fos (Figure 2E) and c-Jun (Figure 2F) in the brain, we expected to find lower levels of major innate pro-inflammatory cytokines in the CNS after PTZ injection. Indeed, C4B-deficient mice could not efficiently upregulate these three cytokines in the brain when compared to WT or C3^–/–^ mice (Figures 4C-E). At the same time, both C3- and C4B-deficient mice had ∼3-fold higher baseline levels of IL-6 in the CNS (Figure 4D). Thus, our data indicate that C4B-deficient mice could not efficiently upregulate genes downstream of IEGs such as neurotrophic factor BDNF and major pro-inflammatory cytokines IL-1β, IL-6, and TNF.

### 3.4. C3- and C4-deficient animals have elevated baseline levels of synaptic marker PSD95

It was established that C4B- and C3-deficient mice had a higher density of axons and a higher number of synapses (Chu et al., 2010; Yilmaz et al., 2021). However, the axonal density and number of synapses (and PSD95-positive post-synaptic densities) were shown to be comparable in the CNS of C3 vs. C4 deficient mice (Gomez-Arboledas et al., 2021; Yilmaz et al., 2021). To confirm this, we compared the number of synaptic and neuronal genes in the brain of WT, C3, and C4B-deficient animals at baseline level and after PTZ injection. We found that both C3- and C4B-deficient mice had ∼1.5-fold higher baseline mRNA level of expression of *PSD95* in the brain (Figure 4F), which was confirmed on a protein level (Supplementary Figure 3). After PTZ injection, mRNA expression of PSD95 was decreased in the brain compared to unmanipulated animals and was not statistically different among WT, C3^–/–^, and C4B^–/–^ experimental groups (Figure 4F). We did not find statistically significant differences for baseline and post-PTZ levels for other tested neuronal genes *Syn1* and *Mecp2* in the brain (not shown). Thus, we found elevated baseline levels of synaptic marker PSD95 on mRNA and protein levels for both C3- and C4B-deficient mice when compared to WT mice, which is likely connected with the higher number of synapses in these animals. In support of our conclusion, it was shown that C3b deposits required for synapse elimination were found to be co-localized with PSD95 (Gomez-Arboledas et al., 2021), while the level of C3 is also reduced in CNS of C4B-deficient mice (Yilmaz et al., 2021).

### 3.5. C4B-, but not C3-, deficient mice have low baseline level of EGR1 protein

**FIGURE 5:**
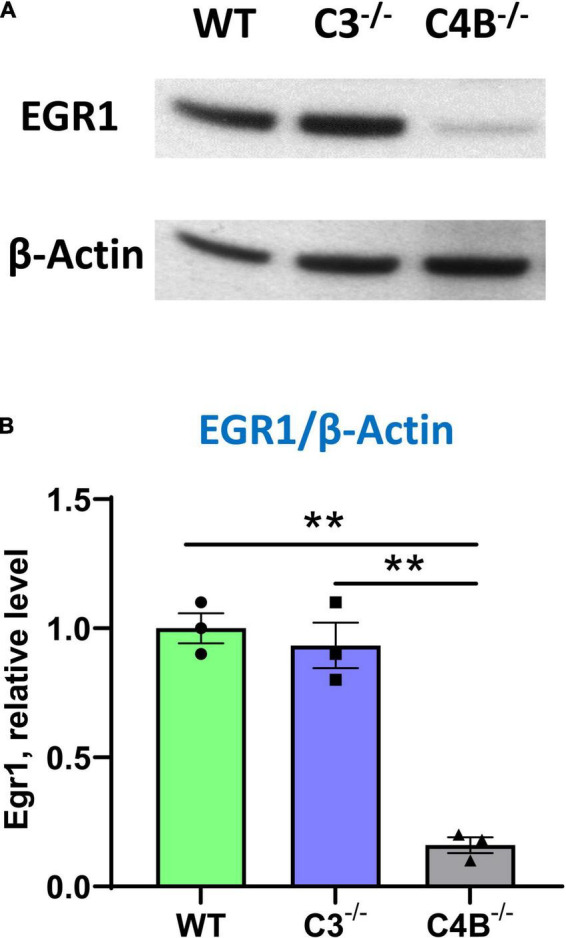
Analysis of the expression of Egr1 on a protein level in the brain cortex of unmanipulated WT, or C3^–/–^, or C4B^–/–^ mice. Expressions of Egr1 and β-Actin were analyzed by western blot as described in Section “2. Materials and methods”. A representative blot for Egr1 [**(A)**, upper image], β-Actin [**(A)**, bottom image], and quantitative analysis of Erg1/βActin ratio **(B)** are shown. In panel **(B)**, mean ± standard error (SE) with overlayed dot plots is shown. The indicated differences were statistically significant, as determined by one-way ANOVA followed by Tukey *post-hoc* test (**p < 0.01 for comparisons between two groups; *n* = 3 mice).

Following our experiment that demonstrated a very low baseline mRNA level of Egr1 in the CNS of unmanipulated C4B^–/–^ mice (Figure 2A), we investigated the baseline level of expression of EGR1 in the brain on a protein level in these animals by western blotting. Quantitative analysis revealed ∼5-fold lower level of ERG1 in the CNS of unmanipulated C4B-deficient mice when compared to WT and C3-deficient animals (Figure 5). Thus, C4B-deficient mice have a low baseline level of Egr1 in the CNS on mRNA and protein levels.

### 3.6. C3-/- and C4B^–/–^ animals have motor function deficiency

**FIGURE 6:**
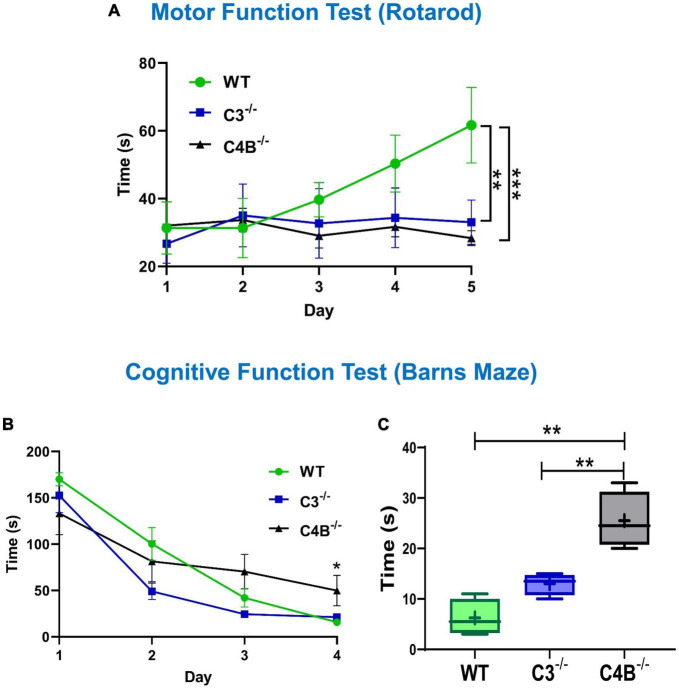
Comparison of motor and cognitive functions of unmanipulated WT, C3-, and C4B-deficient mice. Unmanipulated WT, or C3^–/–^, or C4B^–/–^ mice were assessed for their performance in Rotarod **(A)** and Barns maze **(B,C)** tests as described in Section “2. Materials and methods”. In panel **(A)**, the time to fall from the rotating rod is shown for WT, or C3^–/–^, or C4B^–/–^ mice in the Rotarod test on day 1, day 2, day 3, day 4, and day 5. The mean ± standard error (SE) of time to fall (y-axis) vs. day of training (x-axis) is shown [C3^–/–^ vs. WT: **p < 0.01, C4^–/–^ vs. WT: ***p < 0.001; two-way ANOVA, C4^–/–^ vs. WT: *F*(4,16) = 13.90; C3^–/–^ vs. WT: *F*(4,16) = 6.439; C4^–/–^ vs. C3^–/–^: not significant; *n* = 3 mice]. In panels **(B,C)**, the latency time is shown for WT, or C3^–/–^, or C4B^–/–^ mice in the Barns maze test during the training period on day 1, day 2, day 3, and day 4 **(B)**, and the final trial on day 5 **(C)**. In panel **(B)**, the mean ± standard error (SE) of latency time (y-axis) vs. day of training (x-axis) is shown. The indicated difference on day 4 was statistically significant, as determined by one-way ANOVA followed by Tukey *post-hoc* tests [*p < 0.05 for comparisons C4^–/–^ vs. WT and C4^–/–^ vs. C3^–/–^ on day 4; *n* = (4 mice × 3 trails) = 12]. In panel **(C)**, the median with 10%/90% percentiles of individual mice are shown in a box and whisker plot with the mean value indicated by “+”. The indicated differences were statistically significant for the final trial on day 5, as determined by one-way ANOVA followed by Tukey *post-hoc* tests [**p < 0.01 for comparisons between two groups; *F*(2,9) = 23.56; *n* = 4 mice].

We hypothesized that a low baseline level of Egr1 in C4B-deficient mice could be associated with behavior changes, which is reflected in the performance in motor and cognitive function tests. We found that both C3- and C4B-deficient mice had significantly worse performance in the Rotarod test during a 5-day training course when compared to WT mice (Figure 6A). Thus, we found that both C3- and C4B-deficient mice had deficiency in their motor functions, which is likely linked to the deficiency in synapse pruning in these animals.

### 3.7. C4B-, but not C3-, deficient mice have cognitive dysfunctions

We further investigated whether a low baseline level of Egr1 in C4B-deficient mice could be associated with worse performance in cognitive function test. When we compared cognitive (memory) functions in the Barns maze test, we found for both the training session on day 4 (Figure 6B) and the final trial on day 5 (Figure 6C) the performance results were statistically significantly worse for C4B^–/–^, but not C3^–/–^ mice when compared to WT animals (Figures 6B, C). Thus, these data indicate that the deficiency in Egr1 expression at the baseline level is associated with cognitive problems in C4B^–/–^ animals.

## 4. Discussion

**FIGURE 7:**
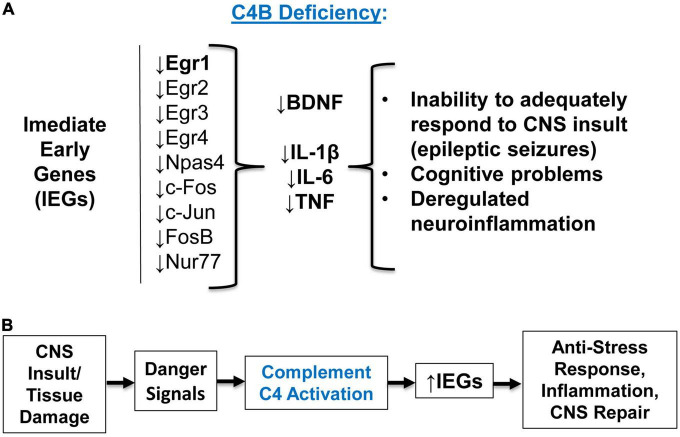
The summary of experimental results and concluding model. **(A)** Summary of the experimental results. C4B deficiency results in a low level of Egr1 in the CNS at the baseline level, and these animals are unable to upregulate Egr1-4, c-Fos, c-Jun, Npas4, and other immediate early genes (IEGs) during epileptic seizures. Inability to upregulate IEGs results in the inability to upregulate downstream target genes BDNF and pro-inflammatory cytokines IL-1β, IL-6, and TNF. Deficiency in IEGs and their downstream targets result in inappropriate responses to CNS insult, injury, or stress conditions. **(B)** Concluding model. Complement C4 activation requirement for IEG upregulation. CNS (or other tissue) insult or infection results in initial tissue damage and release of proper “danger signals”, which lead to activation of complement C4. Activated C4 induces expression of IEGs and their downstream genes required for anti-stress, inflammatory, and tissue repair responses.

In this study, we compared the outcome of PTZ-induced epileptic seizures and behavior deviations in C3^–/–^ vs. C4B^–/–^ mutant mice in comparison with non-mutant WT animals. The rationale for this study was to identify a new role of C4 beyond its upstream activator of C3. We found that *C3*- and *C4B*-deficient mice shared several phenotypical features: both mutants have elevated levels of synaptic marker PSD95 (Figure 4F, Supplementary Figure 3), an elevated baseline level of pro-inflammatory cytokine IL-6 (Figure 4D), and problems in motor functions (Figure 6A). These data support the currently dominating paradigm that C4 acts as an upstream activator of C3 in classical pathway that are involved in CNS functions such as synapse pruning. In the frame of this paradigm, the phenotype of C4- and C3-deficient animals is identical or at least very similar. However, we also found that C4B^–/–^ has many phenotypical features very different from C3^–/–^ animals. Specifically, C4B^–/–^, but not C3^–/–^ mutants, were highly sensitive to PTZ-induced epileptic seizures leading to very severe seizures with 100% mortality rate in a convulsant dose of PTZ (80 mg/kg) and a 60% mortality even in case of subconvulsant dose of PTZ (50 mg/kg) (Figure 1; Table 2). The high susceptibility of C4B-deficient animals to PTZ was associated with the inability of these mutants to upregulate multiple IEGs (Egrs1-4, c-Fos, c-Jun, FosB, Nur77, and Npas4) and their downstream targets such as neurotrophic factor BDNF and cytokines IL-1β, IL-6 and TNF (Figures 2–4). In addition, C4B^–/–^ mice had a low baseline level of Egr1 in the CNS on mRNA and protein levels (Figures 2, 5), which is related to cognitive problems in these animals (Figures 6B, C). We believe that the inability to upregulate IEGs and their downstream targets leads to the poor outcome of epileptic seizures and possibly other conditions such as stress or neuronal injury as summarized in Figure 7A.

Thus, our data indicate that C4B, but not C3, play an important role in the CNS to facilitate upregulation of IEG expression during CNS insults such as seizures (and likely other stress conditions), and the expression of these IEGs and their downstream targets (e.g., BDNF) is very important to cope with neuropathological conditions. The results from our study make us believe that poor ability to deal with stresses, injuries, or CNS insults (e.g., failure upregulate multiple IEGs or maintain Egr1 at baseline level) in case of C4B functional deficiency could potentially predispose to the development of certain neurologic conditions such as SZ and ASD, where stress was shown to be an aggravating factor (Walker et al., 2008; Theoharides and Kavalioti, 2019). In the case of epilepsy, a traumatic brain injury could be a such factor that predisposes to the development of seizures (Englander et al., 2014). Moreover, the baseline level of certain IEGs such as Egr1 in the CNS of C4B-deficient individuals could lead to insufficient axonal growth and synapse formation during CNS development in humans and subsequent cognitive problems and behavior deviations. Recently it was demonstrated that iPSC-derived astrocytes of patients with ASD express low levels of C4 (the study did not discriminate C4A and C4B isoforms) on both mRNA and protein levels (Mansur et al., 2021). These low baseline levels of C4 in the CNS of these subjects could potentially lead to low Egr1 levels in ASD subjects, as we observed in C4B-deficient mice. Studies in mouse and zebrafish models demonstrated an important role for Egr1 in social behavior and the formation of proper neuronal circuits (Tallafuss et al., 2022), while in humans Egr1 was shown to be connected with stress-related mood disorders, schizophrenia, and depression (Duclot and Kabbaj, 2017). Patients with certain systemic autoimmune diseases such as systemic lupus erythematosus (SLE) exhibit neuropsychiatric symptoms and cognitive problems known as “lupus fog” (Monahan et al., 2021). At the same time SLE patients have low level of C4 (Wang and Liu, 2021), which might be connected with these neurologic symptoms. It was also found recently that Egr1 gene is related to SLE pathology (Udhaya Kumar et al., 2020). Thus, our study provides the missing link between C4 and Erg1 in the pathogenesis of aforementioned disorders.

Egr1 belongs to the family of IEGs, which is the family of genes that can become transcribed immediately using an existing pool of kinases or other secondary messengers within minutes after stimulation without the need of the synthesis of new proteins (Pérez-Cadahía et al., 2011). Multiple stimuli induce expression of IEGs that include trophic factors downstream of receptor tyrosine kinases (RTKs, e.g., EGFR, TrkA, TrkB), calcium channels (e.g., NMDAR), neurotransmitters (glutamine, dopamine), and viral infection (Bahrami and Drabløs, 2016; Minatohara et al., 2016; Gallo et al., 2018). In the CNS, c-Fos, Egr1, and Arc are used as a markers of neuronal activity in the context of memory formation and development of multiple psychiatric disorders such as SZ and ASD (Minatohara et al., 2016; Gallo et al., 2018). It was previously shown that multiple IEGs, including Egr1, Egr4, c-Fos, c-Jun, Naps4, Nur77, and Arc were upregulated in *in vivo* animal (from rodents to primates) and in *in vitro* models of epilepsy, as was confirmed in our study (Kiessling and Gass, 1993; Barros et al., 2015; Kalinina et al., 2022; Rienecker et al., 2022). However, in contrast to earlier studies that used *in situ* hybridization, repetitive stimulations with PTZ, or using the earliest time-point at 30 min, we used more sensitive quantitative real-time PCR, which allowed us to detect upregulation of ten IEGs within a 9 ± 1 min period post-PTZ injections to ensure that all tested animals are alive. We were very strict in our kinetic time-point since C4B^–/–^ mice died 8–12 post PTZ and we had to select only alive animals for gene expression analysis. Using this technique, we also showed defective upregulation of cytokines (IL-1β, IL-6, TNF) and tissue repair factors (BDNF) in C4B-deficient mice, but not GABA_*A*_ receptor subunits, which probably require more time for upregulation within a 9-min period. Neuronal c-Fos, Egr1, and Arc have been also implicated in learning, memory, and long-term potentiation (LTP) (Gallo et al., 2018), and we found a significant reduction of Egr1 in normal CNS and during seizures in C4B-deficient mice. Thus, there is a possible link between memory function and expression of Egr1, as we also found in our study, which demonstrates the correlation between a low level of baseline Egr1 expression and performance in the Barns maze test. However, our study shows only association of behavior problems in C4-deficient mice with low level of Erg1 expression in the CNS.

The exact mechanism of connection of IEG expression and C4 activation in the CNS is currently not clear. Among known classical complement receptors (CR), only type 1 complement receptor (CR1/CD35) was shown to bind both C4b and C3b fragments of cleaved C4 and C3 using two different domains for C4b and C3b, and this receptor is associated with the pathogenesis of certain autoimmune diseases (Khera and Das, 2009). Thus, CR1 was the only documented receptor for C4. Despite CR1 being mainly expressed in erythrocytes, B cells, macrophages, and granulocytes (Khera and Das, 2009), the new type 1 complement receptor CSMD1 was found to be highly expressed in the CNS and epithelial cells (Kraus et al., 2006). CSMD family consists of CSMD1, CSMD2, and CSMD3 members, and CSMD1 is the most well-studied member (Ermis Akyuz and Bell, 2022). Similar to CR1, CSMD1 was shown to bind C4b and C3b to promote their degradation by Factor I (Escudero-Esparza et al., 2013), and it is highly expressed in the CNS in 80% of GABAR-positive synapses and 40% of NMDAR-positive synapses (Baum et al., 2020). CSMD1 and CSMD2 polymorphisms were associated with SZ (Håvik et al., 2011; Rose et al., 2013), while CSMD3 was associated with both SZ and ASD (Mizukami et al., 2016). It was shown that CSMD1 could bind and inhibit RTKs such as EGFR (potent inducer of IEGs) and CSMD1 can also modulate TGFβ receptor signaling (Gialeli et al., 2021; Ermis Akyuz and Bell, 2022). Although both EGFR and TGFβRs are expressed in the CNS (Dobolyi et al., 2012; Romano and Bucci, 2020), neuronal cells have other important RTKs such as TrkA and TrkB neurotrophic receptors, that bind BDNF and NGF, respectively (Haddad et al., 2017). There is the possibility that CSMD1 or other members of this family could bind to certain neuronal RTKs and inhibit them in normal CNS. Upon C4 cleavage into the C4b fragment during epileptic seizures or other stress conditions CSMD1 could bind to C4b and no longer inactivate RTKs and the level of IEGs is elevated. Alternatively, CSMDs could also send intracellular signals to induce IEG expression. Further studies will allow us to further elucidate the exact mechanisms of induction of neuronal IEGs by C4. In support of our hypotheses and assumptions stated above, we have previously found that the blood-brain barrier becomes compromised during PTZ-induced seizures, which is accompanied by rapid activation of platelets and blood coagulation cascade leading to activation of complement as well (Dukhinova et al., 2018; Kopeikina et al., 2020; Kopeikina and Ponomarev, 2021).

It is also not clear why C4 plays a more important role in the process of IEG induction when compared to C3. We believe this happens because, in the case of C4 deficiency, the activation of both C4 and C3 is affected (both C4b and C3b fragments are missing), while in the case of C3 deficiency, activation of C4 is still intact (C4b fragments are still present). This is especially important if we remember the fact that CR1 receptors (CR1/CD35 and CSMDs) bind both C4b and C3b (Khera and Das, 2009; Ermis Akyuz and Bell, 2022). In support of this assumption, it was found that the level of C4 remains high in the CNS of C3-deficient mice, while C4B^–/–^ mice have a decrease in both C3 and C4 in the CNS (Yilmaz et al., 2021). In the periphery, we also found in our study that the level of C4 was normal in the serum of C3-deficient mice (Supplementary Figure 2), while C4B^–/–^ had a lower level of C3 activation when compared to WT mice as determined by the intensity of small C3α fragments (within 37–50 kDa range) in western blot analysis of mouse serum (Supplementary Figure 1). We assume that during blood coagulation C4 also play an important role and we see lower level of C3 cleavage in serum samples of C4B-deficient mice (Supplementary Figure 1A). Giving the fact that C4 and C3 have structural similarity (C4 is a paralog for C3 sharing ∼30% of sequence homology) (Blanchong et al., 2001; Mortensen et al., 2015), and both bind to CRs of type 1, it is not very surprising that C4 genetic deficiency in mice has a stronger influence on phenotype when compared to C3 deficiency. In our study we did not analyze the presence of activated C4 in the brain of mice after PTZ administration. Most likely C4 is cleaved in the CNS during seizures by Factor I into iC4b, then into C4d and C4c (Wang and Liu, 2021), and C4d could be deposited on injured neuronal cells. Future studies will investigate C4 depositions in the CNS after seizures.

We believe that initiation/enhancement of IEGs by C4 is a general mechanism of the response of many cell types to pathogen invasion and/or tissue injury where C4 play a key role as a main opsonin of damaged or infected cells (Mortensen et al., 2015). A recent study indicates that a low level of C4B is associated with a high level of mortality after acute myocardial infarction (Blaskó et al., 2008). Given the important role of Egr1 in the regulation of myocardial cell injury (Khachigian, 2016), it is highly likely that expression of Egr1 and other IEGs is enhanced by C4 complement activation during cardiovascular pathological conditions. During viral infection, many viruses specifically inactivate C4, most likely avoiding the upregulation of IEGs in infected cells (Wang and Liu, 2021). Finally, in certain autoimmune diseases such as SLE, C4, and CR1 were shown to play an important role (Blanchong et al., 2001; Zhou et al., 2021).

Our concluding model is shown in Figure 7B. According to this model, CNS (or other tissues) insults result in initial tissue injury when complement activation occurs in response to “danger signals” as was proposed recently (Reis et al., 2019). Activation of complement C4 results in the upregulation of IEGs and proper downstream anti-stress, inflammatory, and tissue repair genes (Figure 7B). Thus, we believe that the connection between C4 and IEG expression could be a new and universal mechanism of cell activation during pathogen invasion and/or tissue injury in the CNS and the periphery.

## Data availability statement

The original contributions presented in this study are included in the article/Supplementary material, further inquiries can be directed to the corresponding author.

## Ethics statement

This animal study was reviewed and approved by the Food Department of the Government of Hong Kong, the Chinese University of Hong Kong, and the City University of Hong Kong Animal Experimentation Ethics Committee.

## Author contributions

TV and EP conceived the study and performed the experiments. TV, RJ, MH, and EP analyzed the data and prepared the manuscript. All authors contributed to the article and approved the submitted version.

## Footnotes

1
http://www.nc3rs.org.uk/arrive-guidelines
