# Supplementary material for: Complement C4-deficient mice have a high mortality rate during PTZ-induced epileptic seizures, which correlates with cognitive problems and the deficiency in the expression of Egr1 and other immediate early genes
Source: Front Cell Neurosci. 2023 May 10;17:1170031. doi: 10.3389/fncel.2023.1170031 (PMC10206007; doi:10.3389/fncel.2023.1170031)
Supplement: Supplementary file 1 [file Data_Sheet_1.PDF]

Supplementary Material

Complement C4-Deficient Mice Have High Mortality Rate during PTZ-Induced Epileptic Seizures, which Correlates with Cognitive Problems and the Deficiency in the Expression of Egr1 and Other Immediate Early Genes

Tatyana Veremeyko[1,2,3], Rongcai Jiang[4,5], Mingliang He[1], and Eugene D. Ponomarev[1,2,3]*

[1]Department of Biomedical Sciences, Jockey Club College of Veterinary Medicine and Life Sciences, City University of Hong Kong, Hong Kong

[2]Department of Biology, School of Sciences and Humanities, Nazarbayev University, Astana, Kazakhstan

[3]Kunming Institute of Zoology, The Chinese Academy of Sciences, and the Chinese University of Hong Kong Joint Laboratory of Bioresources and Molecular Research of Common Diseases, Kunmin, China

[4]Department of Neurosurgery, Tianjin Medical University General Hospital, Tianjin, China

[5]Tianjin Neurological Institute, Key Laboratory of Post Neuro-Injury Neuro-Repair and Regeneration in Central Nervous System, Ministry of Education and Tianjin City, Tianjin, China

*** Correspondence:** Eugene D. Ponomarev: eugene.ponomarev@cityu.edu.hk; eugene.ponomarev@nu.edu.kz

Supplementary Figures: Supplementary Figures 1-3

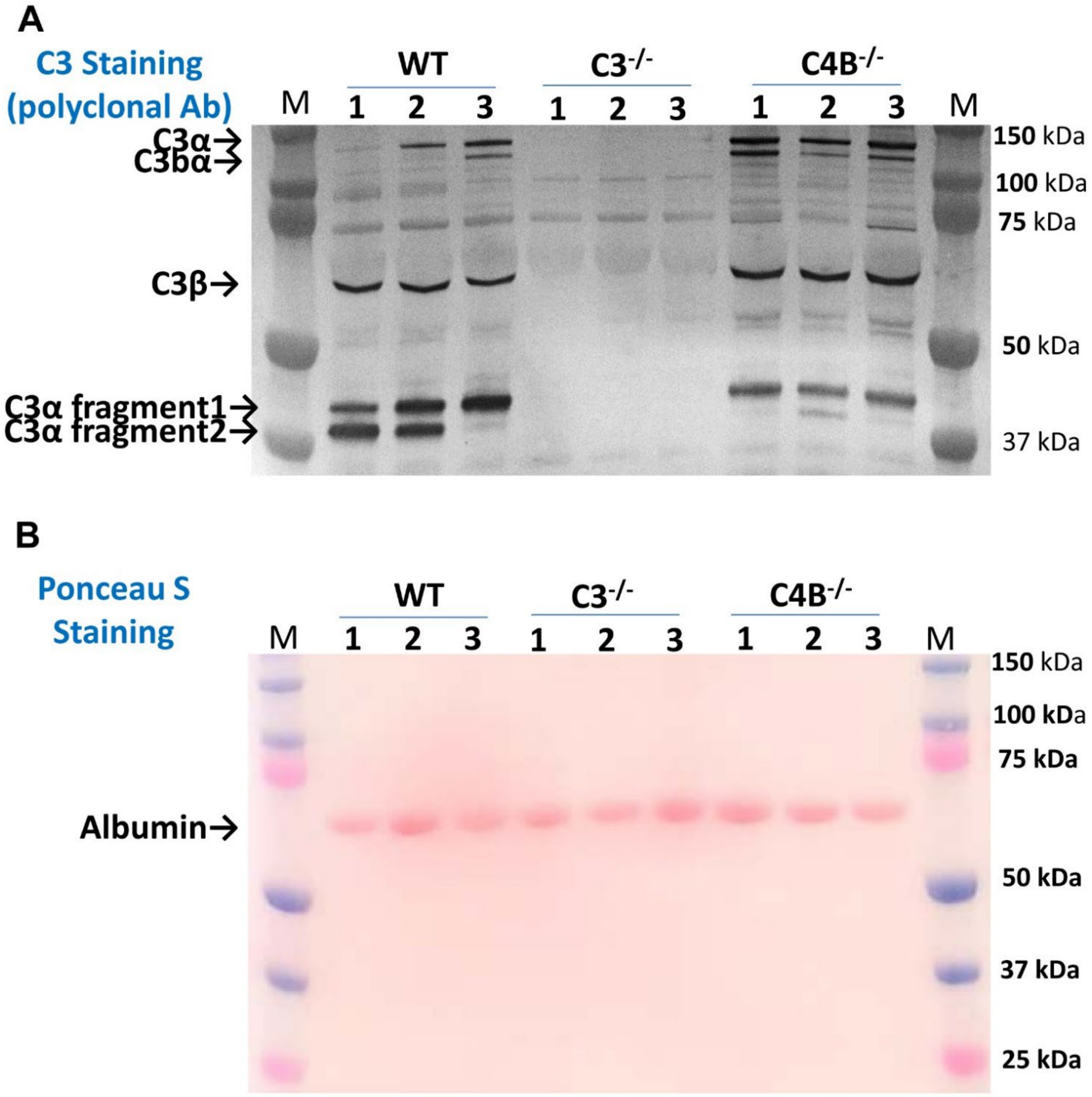

<u>Supplementary Figure 1</u>. Analysis of C3 protein content in the serum of unmanipulated WT, C3$^{-/-}$, and C4B$^{-/-}$ mice. Serum was obtained from alive animals and analyzed for C3 concentration by <u>Western blotting</u> (**A**) and general protein content using <u>Ponceaus S</u> staining (**B**) as described in *Materials and Methods*. For C3 detection, we used polyclonal antibodies that recognize intact α- and β- chains of C3 (C3α and C3β) and C3α fragments of activated C3 (longer C3bα fragment (>100 kDa) and shorter C3α fragment 1 and C3α fragment 2 (37-50 kDa)) as indicated by arrows [n=3 mice for each group].

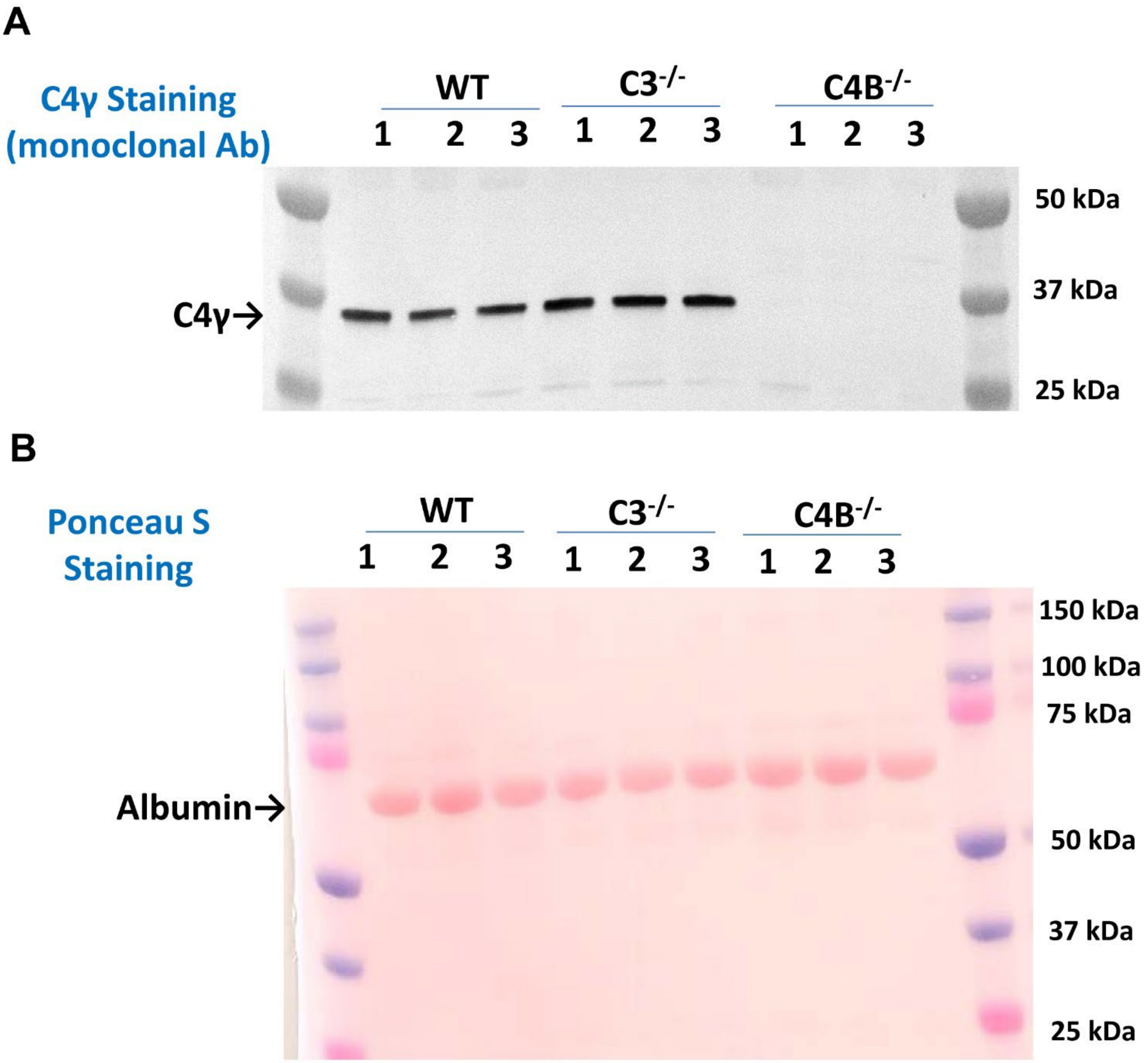

<u>Supplementary Figure 2</u>. Analysis of C4 protein content in the serum of unmanipulated WT, C3$^{-/-}$, and C4B$^{-/-}$ mice. Serum was obtained from alive animals and analyzed for C4 concentration by <u>Western blotting</u> (**A**) and general protein content using <u>Ponceau S</u> staining (**B**) as described in *Materials and Methods*. For C4 detection we used monoclonal antibodies that recognize the γ-chain of C4 (shown as **C4γ**), which is not modified (cleaved) during C4 activation [n=3 mice for each group].

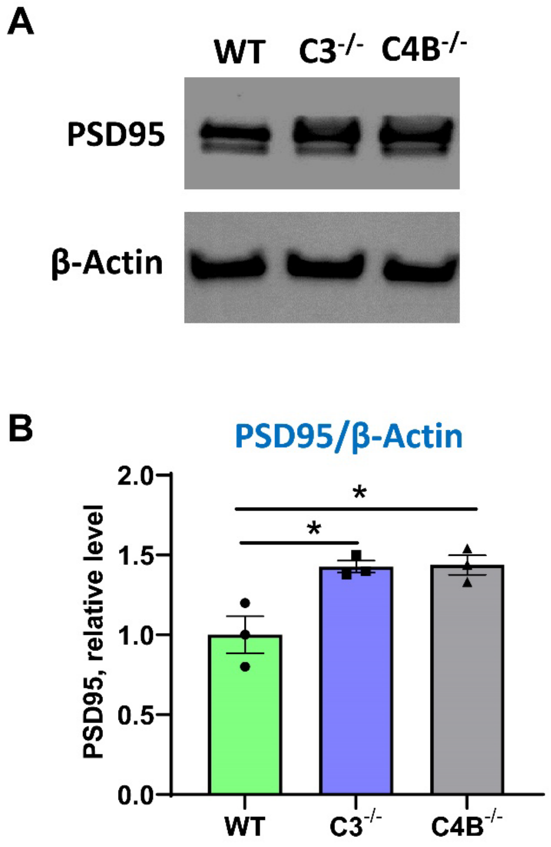

<u>Supplementary Figure 3</u>. Analysis of the expression of PSD95 on a protein level in the brain cortex of unmanipulated WT, or $C3^{-/-}$, or $C4B^{-/-}$ mice. Expressions of PSD95 and β-Actin were analyzed by western blot as described in *Materials and Methods*. A representative blot for PSD95 (**A**, upper image), β-Actin (**A**, bottom image), and quantitative analysis of PSD95/βActin ratio (**B**) are shown.

In (**B**), mean ± standard error (SE) with overlayed dot plots is shown. The indicated differences were statistically significant, as determined by one-way ANOVA followed by Tukey posthoc test [*, $p < 0.05$ for comparisons between two groups; $n = 3$ mice, $F (2, 6) = 10.14$, $p = 0.0119$].
